# Bis(triethyl­ammonium) bis­(μ-pyrazine-2,3-dithiol­ato)bis­(pyrazine-2,3-dithio­lato)diferrate(III) methanol disolvate

**DOI:** 10.1107/S1600536808041949

**Published:** 2008-12-17

**Authors:** Toshiki Yamaguchi, Shigeyuki Masaoka, Ken Sakai

**Affiliations:** a Department of Chemistry, Faculty of Science, Kyushu University, Hakozaki 6-10-1, Higashi-ku, Fukuoka 812-8581, Japan

## Abstract

In the title compound, (C_6_H_16_N)_2_[Fe_2_(C_4_H_2_N_2_S_2_)_4_]·2CH_4_O, the [Fe^III^(pdt)_2_]^−^ anion (pdt is pyrazine-2,3-dithiol­ate) forms a centrosymmetric dimer supported by two Fe^III^—S bonds [Fe—S = 2.4787 (4) Å]. In the crystal structure, dimers form a one-dimensional stack along the *b* axis via π–π stacking inter­actions, the inter­planar separation between adjacent dimers being 3.51 (2) Å. The methanol solvent mol­ecule is involved in two hydrogen bonds in which the hydroxyl group acts as a hydrogen-bond donor to the N atom of a pdt ligand and the O atom acts as an acceptor for the NH group of the triethyl­ammonium cation.

## Related literature

For background information, see: Adams (1990 ▶); Frey (2002 ▶); Georgakaki *et al.* (2003 ▶); Gloaguen *et al.* (2001 ▶); Liu *et al.* (2005 ▶); Nicolet *et al.* (1999 ▶); Peters *et al.* (1998 ▶); Sakata (2000 ▶); Sun *et al.* (2005 ▶); Trasatti (1972 ▶); Yamaguchi *et al.* (2008 ▶). For other iron(III)–dithiol­ene complexes, see: Simao *et al.* (2006 ▶); Yamaguchi *et al.* (2008 ▶). For the synthesis, see: Ribas *et al.* (2004 ▶).
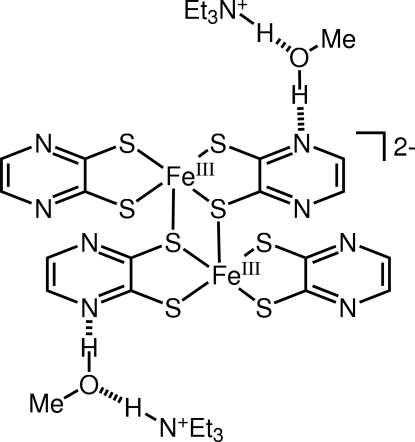


## Experimental

### 

#### Crystal data

(C_6_H_16_N)_2_[Fe_2_(C_4_H_2_N_2_S_2_)_4_]·2CH_4_O
*M* *_r_* = 949.04
Monoclinic, 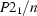

*a* = 14.2375 (15) Å
*b* = 7.9500 (8) Å
*c* = 17.7456 (18) Å
β = 95.293 (1)°
*V* = 2000.0 (4) Å^3^
*Z* = 2
Mo *K*α radiation
μ = 1.19 mm^−1^
*T* = 100 (2) K
0.33 × 0.18 × 0.16 mm

#### Data collection

Bruker SMART APEX CCD-detector diffractometer
Absorption correction: multi-scan (*SADABS*; Sheldrick, 1996 ▶) *T*_min_ = 0.695, *T*_max_ = 0.831
10059 measured reflections
4048 independent reflections
3824 reflections with *I* > 2σ(*I*)
*R*_int_ = 0.013

#### Refinement

*R*[*F* ^2^ > 2σ(*F* ^2^)] = 0.021
*wR*(*F* ^2^) = 0.054
*S* = 1.07
4048 reflections
240 parameters
H-atom parameters constrained
Δρ_max_ = 0.40 e Å^−3^
Δρ_min_ = −0.19 e Å^−3^

### 

Data collection: *APEX2* (Bruker, 2007 ▶); cell refinement: *SAINT* (Bruker, 2007 ▶); data reduction: *SAINT*; program(s) used to solve structure: *SHELXS97* (Sheldrick, 2008 ▶); program(s) used to refine structure: *SHELXL97* (Sheldrick, 2008 ▶); molecular graphics: *KENX* (Sakai, 2004 ▶); software used to prepare material for publication: *SHELXL97*, *TEXSAN* (Molecular Structure Corporation, 2001 ▶), *KENX* and *ORTEPII* (Johnson, 1976 ▶).

## Supplementary Material

Crystal structure: contains datablocks global, I. DOI: 10.1107/S1600536808041949/lh2741sup1.cif

Structure factors: contains datablocks I. DOI: 10.1107/S1600536808041949/lh2741Isup2.hkl

Additional supplementary materials:  crystallographic information; 3D view; checkCIF report

## Figures and Tables

**Table 1:** Hydrogen-bond geometry (Å, °)

*D*—H⋯*A*	*D*—H	H⋯*A*	*D*⋯*A*	*D*—H⋯*A*
O1—H24⋯N3^i^	0.84	1.99	2.8014 (17)	163
N5—H20⋯O1	0.93	1.86	2.7880 (17)	172

Symmetry code: (i) 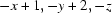.

**Fig. 1.**
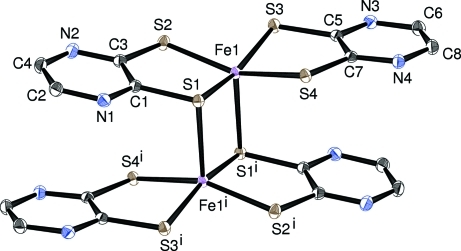
The molecular structure of a [FeIII(pdt)2]22-, dimer, showing the atom-labeling scheme [symmetry code: (i) 1 - x, 2 - y, -z]. Hydrogen atoms are omitted for clarity. Thermal ellipsoids are drawn at the 50% probability.

**Fig. 2.**
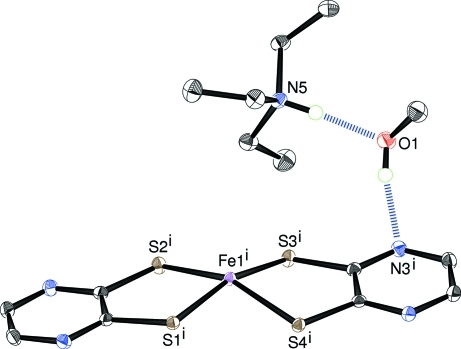
The molecular structure of (Et3NH)[FeIII(pdt)2].MeOH, showing how the hydrogen bonds are formed [symmetry code: (i) 1 - x, 2 - y, -z]. Hydrogen atoms except for those of N—H and O—H are omitted for clarity. Thermal ellipsoids are drawn at the 50% probability.

**Fig. 3.**
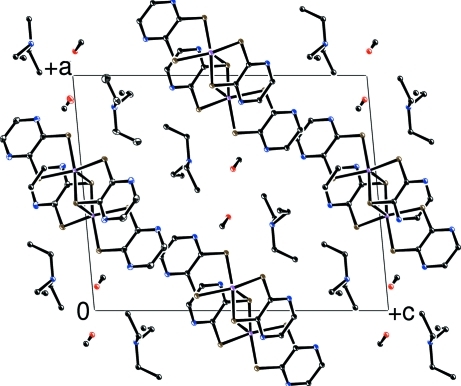
A view down the b axis, showing the manner how the anions stack along the b axis to give one-dimensional chains. Hydrogen atoms are omitted for clarity. Thermal ellipsoids are drawn at the 50% probability.

**Fig. 4.**
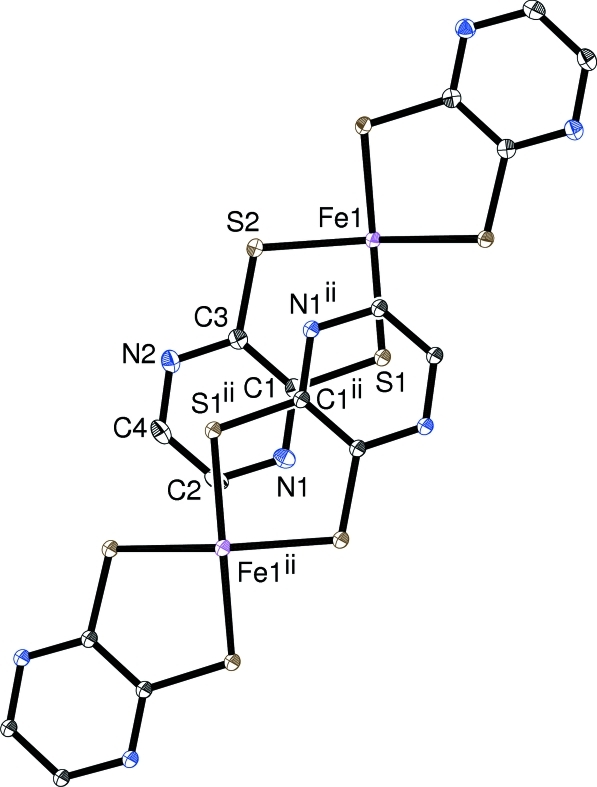
A view perpendicular to the plane defined by atoms S1—S2/C1—C4/N1—N2 which has a weak π-stack to the plane defined by atoms S1ii, C1ii, and N1ii [Symmetry code: (ii) 1 - x, 1 - y, -z]. Hydrogen atoms are omitted for clarity. Thermal ellipsoids are drawn at the 50% probability.

## supplementary crystallographic information

### Comment

The Fe_2_S_2_ clusters (*i.e.*, H-clusters) in Fe-only hydrogenases (FeHases) are known to be highly active as catalysts towards H_2_-evolving (HE) reaction (Adams, 1990; Peters *et al.*, 1998; Nicolet *et al.*, 1999; Frey, 2002). In contrast, metal iron itself exhibits much lower catalytic activity toward HE reaction than does platinum (Trasatti, 1972; Sakata, 2000). A large variety of structural and functional models of FeHases have been developed and their HE activities have been evaluated so far (Gloaguen *et al.*, 2001; Georgakaki *et al.*, 2003; Liu *et al.*, 2005; Sun *et al.*, 2005). On the other hand, an air-stable di-iron complex with a bio-relevant Fe_2_(µ-S)_2_ core, [Fe^III^(mnt)_2_]_2_^2-^ (mnt = maleonitriledithiolate), was found to serve as an electrode catalyst towards HE reaction in aqueous media (Yamaguchi *et al.*, unpublished results). In the present study, [Fe^III^(pdt)_2_]_2_^2-^ has been synthesized to develop the more highly effective HE catalysts with a bio-relevant Fe_2_(µ-S)_2_ core. The pdt ligand has been selected to examine the effect of introducing *N*(imine) donor in close proximity to the active center of the HE reaction. The HE activity of (I) will be separately reported elsewhere (Yamaguchi *et al.*, unpublished results).

The [Fe^III^(pdt)_2_]^-^ anions form a dimer in the crystal with an inversion center located at the center of the dimer (Figure 1). The monomer-monomer association is supported by two crystallographically equivalent Fe^III^—S bonds [Fe1—S1^i^ = 2.4787 (4) Å; symmetry code: (i) 1 - *x*, 2 - *y*, -*z*]. This structural feature well resembles those observed for other iron(III)-dithiolene complexes as follows. The above intermonomer Fe—S distance is quite comparable to those reported for (Ph_4_As)_2_[Fe^III^(qdt)_2_]_2_ (qdt = quinoxaline-2,3-dithiolate) [Fe—S = 2.4884 (13) Å] (Simao *et al.*, 2006) and [Fe^II^(15-crown-5)(OH_2_)_2_][Fe^III^(mnt)_2_]_2_^.^2(15-crown-5) [Fe—S = 2.4715 (9) and 2.4452 (9) Å] (Yamaguchi *et al.*, 2008). The Fe^III^ ion is considered to have a distorted square pyramidal stereochemistry; the Fe^III^ ion is ligated by four sulfur atoms with shorter Fe—S distances [2.2264 (4)–2.2367 (4) Å] and axially ligated by a sulfur atom from the adjacent monomer with a longer Fe—S distance [2.4787 (4) Å]. Atom Fe1 is shifted out of the least-squares plane defined with four atoms S1—S4 by 0.3719 (3) Å, even though the four-atom r.m.s. deviation given in the calculation was 0.0650 Å.

The methanol molecule is stabilized with two different types of hydrogen bonding interactions (Figure 2). One type of hydrogen bond is formed between the hydroxyl unit of methanol and the nitrogen atom of pdt [O1—N3^i^ = 2.8014 (17) Å; symmetry code: (i) 1 - *x*, 2 - *y*, -*z*]. The other is formed between the N—H group of the triethylammonium cation and the oxygen atom of methanol [O1—N5 = 2.7880 (17) Å].

Finally, the anion forms a one-dimensional stack along the *b* axis (Figure 3). The stack of anions is stabilized with a π-π interaction formed between two adjacent pdt moieties. As shown in Figure 4, a set of atoms S1—S2/C1—C4/N1—N2 and that of atoms S1^ii^, C1^ii^, and N1^ii^ contribute to the π-π association at each interdimer association [symmetry code: (ii) 1 - *x*, 1 - *y*, -*z*]. The interplanar separation is calculated as 3.51 (2) Å based on the average shift of atoms S1^ii^, C1^ii^, and N1^ii^ from the best plane defined by atoms S1—S2/C1—C4/N1—N2. An important short contact at this geometry is C1—C1^ii^ = 3.493 (3) Å [symmetry code: (ii) 1 - *x*, 1 - *y*, -*z*].

### Experimental

Compound (I) was prepared as follows. Pyrazine-2,3-dithiol was prepared as previously described (Ribas *et al.*, 2004). To a suspension of pyrazine-2,3-dithiol (0.146 g, 1.0 mmol) and triethylamine (0.42 ml, 3.0 mmol) in dry tetrahydrofuran (THF, 50 ml) was slowly added a solution of FeCl_3_^.^6H_2_O (0.137 g, 0.5 mmol) in dry THF (5 ml) under Ar atmosphere. Although a dark-brown solid immediately precipitated, the suspension was stirred at room temperature for 18 h. The dark-brown solid deposited was collected by filtration and washed with THF. The crude material was re-dissolved in a minimum amount of methanol followed by filtration for the removal of insoluble materials. Standing of the filtrate at room temperature for several days afforded the black needles of (I), which were collected by filtration, washed with cold methanol, and dried *in vacuo*. Yield: 0.098 g (41%). Single crystals of (I) suitable for X-ray crystallography were grown by slow diffusion of diethyl ether into a methanol solution of (I). Analysis calculated for C_30_H_48_Fe_2_N_10_O_2_S_8_: C, 37.97; H, 5.10; N, 14.76. Found: C, 38.04; H, 4.98; N, 15.04. IR (ν, cm^-1^): 3028 (w), 2672 (w), 1531 (*m*), 1449 (*m*), 1416 (*m*), 1394 (*m*), 1320 (*s*), 1287 (*m*), 1196 (w), 1175 (*m*), 1144 (*s*), 1064 (*m*), 1045 (*m*), 1005 (*m*), 837 (*m*), 825 (*s*), 791 (*m*), 485 (*m*), 475 (*m*), 449 (*s*), 421 (*m*).

### Refinement

All H atoms were placed in idealized position (ring C—H = 0.95 Å, methyl C—H = 0.98 Å, methylene C—H = 0.99 Å, hydroxyl O—H = 0.84 Å and tertiary N—H = 0.93 Å), and included in the refinement in a riding-model approximation, with *U*_iso_(H) = 1.2U_eq_ (ring C, methylene C and tertiary N) and *U*_iso_(H) = 1.5U_eq_ (methyl C and hydroxyl O). In the final Fourier map, the highest peak was located 0.99 Å from atom S4. The deepest hole was located 0.53 Å from atom Fe1.

### Crystal data

**Table:**

(C_6_H_16_N)_2_[Fe_2_(C_4_H_2_N_2_S_2_)_4_]·2CH_4_O	*F*(000) = 988
*M**_r_* = 949.04	*D*_x_ = 1.576 Mg m^−^^3^
Monoclinic, *P*2_1_/*n*	Mo *K*α radiation, λ = 0.71073 Å
Hall symbol: -P 2yn	Cell parameters from 7956 reflections
*a* = 14.2375 (15) Å	θ = 2.3–27.5°
*b* = 7.9500 (8) Å	µ = 1.19 mm^−^^1^
*c* = 17.7456 (18) Å	*T* = 100 K
β = 95.293 (1)°	Needles, black
*V* = 2000.0 (4) Å^3^	0.33 × 0.18 × 0.16 mm
*Z* = 2	

### Data collection

**Table:**

Bruker SMART APEX CCD-detector diffractometer	4048 independent reflections
Radiation source: rotating anode with a mirror focusing unit	3824 reflections with *I* > 2σ(*I*)
graphite	*R*_int_ = 0.013
φ and ω scans	θ_max_ = 26.4°, θ_min_ = 2.3°
Absorption correction: multi-scan (*SADABS*; Sheldrick, 1996)	*h* = −16→17
*T*_min_ = 0.695, *T*_max_ = 0.831	*k* = −9→9
10059 measured reflections	*l* = −22→14

### Refinement

**Table:**

Refinement on *F*^2^	Primary atom site location: structure-invariant direct methods
Least-squares matrix: full	Secondary atom site location: difference Fourier map
*R*[*F*^2^ > 2σ(*F*^2^)] = 0.021	Hydrogen site location: inferred from neighbouring sites
*wR*(*F*^2^) = 0.054	H-atom parameters constrained
*S* = 1.07	*w* = 1/[σ^2^(*F*_o_^2^) + (0.0258*P*)^2^ + 1.2723*P*] where *P* = (*F*_o_^2^ + 2*F*_c_^2^)/3
4048 reflections	(Δ/σ)_max_ = 0.001
240 parameters	Δρ_max_ = 0.40 e Å^−^^3^
0 restraints	Δρ_min_ = −0.19 e Å^−^^3^

### Special details

**Table:**

Experimental. The first 50 frames were rescanned at the end of data collection to evaluate any possible decay phenomenon. Since it was judged to be negligible, no decay correction was applied to the data.
Geometry. All e.s.d.'s (except the e.s.d. in the dihedral angle between two l.s. planes) are estimated using the full covariance matrix. The cell e.s.d.'s are taken into account individually in the estimation of e.s.d.'s in distances, angles and torsion angles; correlations between e.s.d.'s in cell parameters are only used when they are defined by crystal symmetry. An approximate (isotropic) treatment of cell e.s.d.'s is used for estimating e.s.d.'s involving l.s. planes.Least-squares planes (*x*,*y*,*z* in crystal coordinates) and deviations from them (* indicates atom used to define plane)4.6834 (0.0016) *x* + 6.8874 (0.0009) *y* - 7.1795 (0.0020) *z* = 7.7711 (0.0010)* 0.0655 (0.0002) S1 * -0.0639 (0.0002) S2 * 0.0645 (0.0002) S3 * -0.0661 (0.0002) S4 0.3719 (0.0003) Fe1Rms deviation of fitted atoms = 0.0650Least-squares planes (*x*,*y*,*z* in crystal coordinates) and deviations from them (* indicates atom used to define plane)5.7097 (0.0038) *x* + 6.0721 (0.0015) *y* - 9.5934 (0.0044) *z* = 7.6443 (0.0017)* 0.0277 (0.0006) S1 * -0.0176 (0.0006) S2 * -0.0098 (0.0012) C1 * -0.0122 (0.0011) C2 * -0.0067 (0.0012) C3 * 0.0190 (0.0011) C4 * -0.0173 (0.0010) N1 * 0.0170 (0.0010) N2 - 3.5344 (0.0008) S1_$2 - 3.4970 (0.0017) C1_$2 - 3.4894 (0.0014) N1_$2Rms deviation of fitted atoms = 0.0170
Refinement. Refinement of *F*^2^ against ALL reflections. The weighted *R*-factor *wR* and goodness of fit *S* are based on *F*^2^, conventional *R*-factors *R* are based on *F*, with *F* set to zero for negative *F*^2^. The threshold expression of *F*^2^ > σ(*F*^2^) is used only for calculating *R*-factors(gt) *etc*. and is not relevant to the choice of reflections for refinement. *R*-factors based on *F*^2^ are statistically about twice as large as those based on *F*, and *R*- factors based on ALL data will be even larger.

### Fractional atomic coordinates and isotropic or equivalent isotropic displacement parameters (Å^2^)

**Table:**

	*x*	*y*	*z*	*U*_iso_*/*U*_eq_	
Fe1	0.404540 (13)	0.93135 (3)	0.023150 (11)	0.01057 (6)	
S1	0.54285 (2)	0.79904 (4)	0.029127 (18)	0.01130 (8)	
S2	0.35609 (2)	0.79099 (5)	−0.082403 (19)	0.01420 (8)	
S3	0.25466 (2)	1.00013 (5)	0.034182 (19)	0.01363 (8)	
S4	0.43700 (2)	0.97513 (4)	0.147331 (18)	0.01193 (8)	
O1	0.89754 (8)	0.60915 (14)	−0.01530 (6)	0.0209 (2)	
H24	0.8767	0.6838	−0.0459	0.031*	
N1	0.61230 (9)	0.57967 (15)	−0.06370 (7)	0.0151 (3)	
N2	0.44848 (9)	0.57864 (16)	−0.16542 (7)	0.0163 (3)	
N3	0.18638 (8)	1.19999 (16)	0.13603 (7)	0.0147 (2)	
N4	0.34506 (9)	1.16211 (16)	0.24079 (7)	0.0141 (2)	
N5	0.86540 (8)	0.70235 (16)	0.13156 (7)	0.0151 (3)	
H20	0.8819	0.6706	0.0841	0.018*	
C1	0.53659 (10)	0.67055 (18)	−0.05203 (8)	0.0122 (3)	
C2	0.60532 (11)	0.48707 (19)	−0.12700 (9)	0.0171 (3)	
H1	0.6571	0.4184	−0.1377	0.021*	
C3	0.45350 (10)	0.67025 (18)	−0.10198 (8)	0.0132 (3)	
C4	0.52528 (11)	0.48836 (19)	−0.17707 (8)	0.0178 (3)	
H2	0.5246	0.4224	−0.2218	0.021*	
C5	0.26017 (10)	1.10755 (18)	0.12000 (8)	0.0124 (3)	
C6	0.19204 (10)	1.27363 (19)	0.20475 (8)	0.0167 (3)	
H3	0.1412	1.3416	0.2179	0.020*	
C7	0.34105 (10)	1.09206 (18)	0.17242 (8)	0.0119 (3)	
C8	0.26953 (10)	1.25302 (19)	0.25642 (8)	0.0161 (3)	
H4	0.2696	1.3047	0.3047	0.019*	
C9	0.92078 (11)	0.5916 (2)	0.18858 (8)	0.0181 (3)	
H5	0.8909	0.5936	0.2368	0.022*	
H6	0.9856	0.6366	0.1985	0.022*	
C10	0.92556 (12)	0.4119 (2)	0.16109 (9)	0.0229 (3)	
H7	0.8615	0.3687	0.1489	0.034*	
H8	0.9583	0.3423	0.2008	0.034*	
H9	0.9599	0.4082	0.1158	0.034*	
C11	0.76030 (10)	0.6761 (2)	0.13087 (9)	0.0197 (3)	
H10	0.7471	0.5538	0.1296	0.024*	
H11	0.7289	0.7259	0.0840	0.024*	
C12	0.71813 (11)	0.7525 (2)	0.19835 (9)	0.0253 (4)	
H13	0.7530	0.7127	0.2451	0.038*	
H12	0.6518	0.7189	0.1977	0.038*	
H14	0.7223	0.8754	0.1958	0.038*	
C13	0.89199 (11)	0.8848 (2)	0.14175 (9)	0.0193 (3)	
H16	0.8950	0.9141	0.1961	0.023*	
H15	0.8428	0.9557	0.1144	0.023*	
C14	0.98672 (13)	0.9219 (2)	0.11225 (10)	0.0283 (4)	
H19	1.0362	0.8578	0.1416	0.042*	
H18	1.0004	1.0425	0.1173	0.042*	
H17	0.9846	0.8894	0.0589	0.042*	
C16	0.85896 (13)	0.4489 (2)	−0.03924 (10)	0.0263 (4)	
H21	0.8600	0.4378	−0.0942	0.040*	
H22	0.7938	0.4406	−0.0260	0.040*	
H23	0.8968	0.3588	−0.0139	0.040*	

### Atomic displacement parameters (Å^2^)

**Table:**

	*U*^11^	*U*^22^	*U*^33^	*U*^12^	*U*^13^	*U*^23^
Fe1	0.01022 (10)	0.01220 (11)	0.00912 (10)	0.00046 (7)	0.00004 (7)	−0.00049 (7)
S1	0.01119 (16)	0.01259 (17)	0.00985 (15)	0.00098 (12)	−0.00040 (12)	−0.00008 (12)
S2	0.01256 (17)	0.01665 (18)	0.01285 (16)	0.00100 (13)	−0.00173 (13)	−0.00337 (13)
S3	0.01063 (16)	0.01853 (19)	0.01144 (16)	0.00053 (13)	−0.00050 (12)	−0.00270 (13)
S4	0.01160 (16)	0.01452 (17)	0.00948 (16)	0.00168 (13)	0.00002 (12)	0.00010 (12)
O1	0.0232 (6)	0.0213 (6)	0.0173 (5)	0.0043 (5)	−0.0036 (4)	−0.0003 (4)
N1	0.0165 (6)	0.0126 (6)	0.0166 (6)	0.0007 (5)	0.0039 (5)	0.0010 (5)
N2	0.0208 (6)	0.0141 (6)	0.0140 (6)	−0.0008 (5)	0.0009 (5)	−0.0018 (5)
N3	0.0138 (6)	0.0149 (6)	0.0156 (6)	−0.0002 (5)	0.0020 (5)	−0.0004 (5)
N4	0.0167 (6)	0.0131 (6)	0.0125 (6)	0.0002 (5)	0.0017 (5)	−0.0004 (5)
N5	0.0136 (6)	0.0187 (7)	0.0124 (6)	0.0002 (5)	−0.0013 (5)	0.0010 (5)
C1	0.0151 (7)	0.0104 (7)	0.0111 (6)	−0.0007 (5)	0.0019 (5)	0.0008 (5)
C2	0.0192 (7)	0.0120 (7)	0.0211 (7)	0.0014 (6)	0.0067 (6)	−0.0012 (6)
C3	0.0156 (7)	0.0109 (7)	0.0132 (6)	−0.0008 (5)	0.0020 (5)	0.0010 (5)
C4	0.0253 (8)	0.0129 (7)	0.0157 (7)	0.0000 (6)	0.0053 (6)	−0.0027 (6)
C5	0.0133 (7)	0.0120 (7)	0.0122 (6)	−0.0016 (5)	0.0023 (5)	0.0011 (5)
C6	0.0164 (7)	0.0158 (7)	0.0183 (7)	0.0017 (6)	0.0043 (6)	−0.0013 (6)
C7	0.0137 (7)	0.0101 (7)	0.0120 (6)	−0.0013 (5)	0.0018 (5)	0.0019 (5)
C8	0.0197 (7)	0.0156 (7)	0.0134 (7)	0.0003 (6)	0.0035 (6)	−0.0017 (5)
C9	0.0147 (7)	0.0233 (8)	0.0155 (7)	0.0015 (6)	−0.0036 (6)	0.0039 (6)
C10	0.0240 (8)	0.0231 (9)	0.0207 (8)	0.0039 (6)	−0.0017 (6)	0.0045 (6)
C11	0.0122 (7)	0.0259 (8)	0.0201 (7)	−0.0001 (6)	−0.0033 (6)	0.0002 (6)
C12	0.0169 (8)	0.0336 (9)	0.0255 (8)	0.0008 (7)	0.0028 (6)	−0.0024 (7)
C13	0.0208 (8)	0.0184 (8)	0.0177 (7)	−0.0012 (6)	−0.0033 (6)	0.0001 (6)
C14	0.0294 (9)	0.0274 (9)	0.0283 (9)	−0.0089 (7)	0.0048 (7)	−0.0002 (7)
C16	0.0303 (9)	0.0248 (9)	0.0228 (8)	0.0009 (7)	−0.0038 (7)	−0.0038 (7)

### Geometric parameters (Å, °)

**Table:**

Fe1—S1	2.2264 (4)	C9—C10	1.513 (2)
Fe1—S3	2.2289 (4)	C11—C12	1.515 (2)
Fe1—S2	2.2341 (4)	C13—C14	1.520 (2)
Fe1—S4	2.2367 (4)	N5—H20	0.9300
Fe1—S1^i^	2.4787 (4)	C2—H1	0.9500
S1—C1	1.7611 (14)	C4—H2	0.9500
S1—Fe1^i^	2.4787 (4)	C6—H3	0.9500
S2—C3	1.7477 (15)	C8—H4	0.9500
S3—C5	1.7415 (14)	C9—H5	0.9900
S4—C7	1.7438 (14)	C9—H6	0.9900
O1—C16	1.436 (2)	C10—H7	0.9800
O1—H24	0.8400	C10—H8	0.9800
N1—C1	1.3297 (19)	C10—H9	0.9800
N1—C2	1.339 (2)	C11—H10	0.9900
N2—C3	1.3372 (19)	C11—H11	0.9900
N2—C4	1.340 (2)	C12—H13	0.9800
N3—C5	1.3341 (19)	C12—H12	0.9800
N3—C6	1.3483 (19)	C12—H14	0.9800
N4—C7	1.3315 (18)	C13—H16	0.9900
N4—C8	1.3456 (19)	C13—H15	0.9900
N5—C13	1.506 (2)	C14—H19	0.9800
N5—C9	1.5079 (18)	C14—H18	0.9800
N5—C11	1.5097 (19)	C14—H17	0.9800
C1—C3	1.411 (2)	C16—H21	0.9800
C2—C4	1.379 (2)	C16—H22	0.9800
C5—C7	1.417 (2)	C16—H23	0.9800
C6—C8	1.378 (2)		
			
C1···C1^ii^	3.493 (3)		
			
S1—Fe1—S3	164.042 (16)	C4—C2—H1	119.0
S1—Fe1—S2	90.420 (15)	N2—C4—H2	118.6
S3—Fe1—S2	88.401 (15)	C2—C4—H2	118.6
S1—Fe1—S4	85.705 (14)	N3—C6—H3	119.1
S3—Fe1—S4	89.299 (14)	C8—C6—H3	119.1
S2—Fe1—S4	157.445 (17)	N4—C8—H4	118.9
S1—Fe1—S1^i^	97.462 (14)	C6—C8—H4	118.9
S3—Fe1—S1^i^	98.369 (15)	N5—C9—H5	109.3
S2—Fe1—S1^i^	101.461 (15)	C10—C9—H5	109.3
S4—Fe1—S1^i^	101.073 (15)	N5—C9—H6	109.3
C1—S1—Fe1	104.91 (5)	C10—C9—H6	109.3
C1—S1—Fe1^i^	100.94 (5)	H5—C9—H6	107.9
Fe1—S1—Fe1^i^	82.538 (14)	C9—C10—H7	109.5
C3—S2—Fe1	104.58 (5)	C9—C10—H8	109.5
C5—S3—Fe1	103.35 (5)	H7—C10—H8	109.5
C7—S4—Fe1	103.82 (5)	C9—C10—H9	109.5
C1—N1—C2	115.52 (13)	H7—C10—H9	109.5
C3—N2—C4	116.15 (13)	H8—C10—H9	109.5
C5—N3—C6	116.71 (12)	N5—C11—H10	108.8
C7—N4—C8	116.39 (12)	C12—C11—H10	108.8
C13—N5—C9	111.84 (11)	N5—C11—H11	108.8
C13—N5—C11	111.85 (12)	C12—C11—H11	108.8
C9—N5—C11	112.41 (12)	H10—C11—H11	107.7
N1—C1—C3	123.08 (13)	C11—C12—H13	109.5
N1—C1—S1	117.51 (11)	C11—C12—H12	109.5
C3—C1—S1	119.40 (11)	H13—C12—H12	109.5
N1—C2—C4	121.97 (14)	C11—C12—H14	109.5
N2—C3—C1	120.42 (13)	H13—C12—H14	109.5
N2—C3—S2	119.00 (11)	H12—C12—H14	109.5
C1—C3—S2	120.58 (11)	N5—C13—H16	109.3
N2—C4—C2	122.84 (14)	C14—C13—H16	109.3
N3—C5—C7	121.06 (13)	N5—C13—H15	109.3
N3—C5—S3	118.93 (11)	C14—C13—H15	109.3
C7—C5—S3	119.98 (11)	H16—C13—H15	108.0
N3—C6—C8	121.84 (14)	C13—C14—H19	109.5
N4—C7—C5	121.71 (13)	C13—C14—H18	109.5
N4—C7—S4	119.13 (11)	H19—C14—H18	109.5
C5—C7—S4	119.14 (11)	C13—C14—H17	109.5
N4—C8—C6	122.20 (13)	H19—C14—H17	109.5
N5—C9—C10	111.80 (12)	H18—C14—H17	109.5
N5—C11—C12	113.75 (12)	O1—C16—H21	109.5
N5—C13—C14	111.52 (13)	O1—C16—H22	109.5
C16—O1—H24	109.5	H21—C16—H22	109.5
C13—N5—H20	106.8	O1—C16—H23	109.5
C9—N5—H20	106.8	H21—C16—H23	109.5
C11—N5—H20	106.8	H22—C16—H23	109.5
N1—C2—H1	119.0		
			
C2—N1—C1—C3	0.5 (2)	Fe1—S3—C5—N3	164.68 (10)
C2—N1—C1—S1	−178.40 (11)	Fe1—S3—C5—C7	−17.12 (12)
Fe1—S1—C1—N1	178.17 (10)	C5—N3—C6—C8	−0.8 (2)
Fe1^i^—S1—C1—N1	93.07 (11)	C8—N4—C7—C5	−2.2 (2)
Fe1—S1—C1—C3	−0.74 (12)	C8—N4—C7—S4	178.97 (11)
Fe1^i^—S1—C1—C3	−85.84 (11)	N3—C5—C7—N4	3.2 (2)
C1—N1—C2—C4	0.9 (2)	S3—C5—C7—N4	−174.98 (11)
C4—N2—C3—C1	0.9 (2)	N3—C5—C7—S4	−177.96 (11)
C4—N2—C3—S2	−179.75 (11)	S3—C5—C7—S4	3.87 (16)
N1—C1—C3—N2	−1.4 (2)	Fe1—S4—C7—N4	−169.57 (10)
S1—C1—C3—N2	177.43 (11)	Fe1—S4—C7—C5	11.55 (12)
N1—C1—C3—S2	179.20 (11)	C7—N4—C8—C6	−0.2 (2)
S1—C1—C3—S2	−1.95 (17)	N3—C6—C8—N4	1.8 (2)
Fe1—S2—C3—N2	−175.86 (10)	C13—N5—C9—C10	−156.73 (13)
Fe1—S2—C3—C1	3.53 (12)	C11—N5—C9—C10	76.48 (16)
C3—N2—C4—C2	0.5 (2)	C13—N5—C11—C12	−52.48 (17)
N1—C2—C4—N2	−1.5 (2)	C9—N5—C11—C12	74.31 (17)
C6—N3—C5—C7	−1.5 (2)	C9—N5—C13—C14	75.40 (15)
C6—N3—C5—S3	176.64 (11)	C11—N5—C13—C14	−157.50 (13)

Symmetry codes: (i) −*x*+1, −*y*+2, −*z*; (ii) −*x*+1, −*y*+1, −*z*.

### Hydrogen-bond geometry (Å, °)

**Table:**

*D*—H···*A*	*D*—H	H···*A*	*D*···*A*	*D*—H···*A*
O1—H24···N3^i^	0.84	1.99	2.8014 (17)	163
N5—H20···O1	0.93	1.86	2.7880 (17)	172

Symmetry codes: (i) −*x*+1, −*y*+2, −*z*.
